# On Medical Reform

**Published:** 1810-03

**Authors:** 


### *To the Editors of the Medical and Phyſical Journal.*

GENTLEMEN,

As your respectable Correspondent of this month, whose Essay bears no signature, has replied with equal candour and liberality to my former observations, I felt a strong desire to comply with his request, and that of many others, by drawing out a short account of the intended Bill for improving the education and practice of future candidates. With this view, I solicited, and readily obtained the approbation of those who have taken an active part in the measures already instituted. I shall therefore proceed, with your leave, to lay the following statement before your numerous readers. I am enabled to execute my design from having been put into possession of a copy, which having been submitted to, and returned from one of the medical corporations, is now lying before me. The Bill provides,

1st. That all members of the curative art shall be registered in the capacity or capacities in which they practice. They are also to put their names and titles on the outer doors of their dwelling houses; by which means, strangers will be able, on emergencies, to find out proper medical help. Persons guilty of false enteries will be liable to heavy penalties.

2dly. "And be it further enacted, that from and after the day , no person *who shall not before that time* have practised or acted as a physician, shall be entitled to be registered or to take out any such certificate as aforesaid, to practice or act as a physician, or shall be entitled or allowed to practice, or act as a physician, for gain or reward, or be entitled or permitted to claim or receive any recompense or remuneration for such his practice, until he shall have taken the degree of a Doctor or Batchelor of Physic, in some British, or Irish, or Foreign University; and shall have obtained a Diploma or Certificate from the proper officer, of having taken such degree, and also shall have been examined by, and shall have obtained a Diploma or authority to practice as a Physician, from some one of the Colleges of Physicians of England, Scotland, or Ireland, respectively, or from the Faculty of Physicians and Surgeons of Glasgow.

"And no person, *who shall not before that time*, have practiced as a Surgeon, shall be entitled to be registered, or to take out any Certificate to practice as a Surgeon, or shall be entitled or allowed to act as a Surgeon, for gain or reward, or to receive any recompense or remuneration for such practice, until he shall have been examined by, and shall have obtained a Diploma or authority to practice as a Surgeon, from some one of the Colleges of Surgeons in England, Scotland, or Ireland, respectively, or from the Faculty of Physicians and Surgeons of Glasgow. "And no person, *who shall not before that time*, have practiced as an Apothecary, or sold Medicines or Drugs, or chemical preparations as an Apothecary, Chemist, or Druggist, shall be entitled to be registered to practice, or act, or shall be entitled or allowed to practice or act as an Apothecary, for gain or reward, or to sell Medicines or Drugs, or chemical preparations as an Apothecary, or as a Chemist and Druggist, until he shall have served an apprenticeship by regular Indentures to some person or persons, actually practising, or acting, or selling, as aforesaid, in the capacity or capacities, in which the person so proposing to practice, or act, or sell, as aforesaid, shall register himself, and take out his Certificate, as aforesaid, nor until he shall have been examined by and shall have obtained a Certificate for the capacity or capacities in which he is to act, or sell, as aforesaid, from the Company of Apothecaries in England, or Ireland, or Scotland, from a Medical Board, to be established by the Commissioners appointed to carry this act into execution.

"And no person, *not having before that time* practised as a Man-midwife, shall be entitled to be registered, to practice and act, or shall be entitled or allowed to act as a Man-midwife, for gain or reward, or to receive any remuneration or recompence for acting as a Man-midwife, unless he shall be a Physician, Surgeon, or Apothecary, and shall have served or acted for twelve calendar months, under some person or persons, practising as a Man-midwife or Men-midwives, and shall have attended, or actually assisted such person or persons, in his or their practice, as a Man-midwife or Men-midwives, and shall have been examined by, and shall have obtained a Certificate from the Board of Midwifery Practitioners, to be appointed by the said Commissioners.

"And be it further enacted, that nothing in this Act shall extend to prevent any person from administering any medicine, or from practising as a bone-setter, or from acting in any other manner in Medicine or Surgery in which *he shall have acted before the passing of this Act*, from from continuing so to practice, without being subject to any penalties for not being registered, and having a Certificate under this Act as a Physician, Surgeon, Man-midwife, or Apothecary, provided that such person shall have obtained, and have a Certificate and Licence continuing in full force, and not withdrawn, from any three or more Magistrates, assembled at any Quarter or General Adjourned or Petty Sessions of the Peace for the County in which he shall practice, and shall record such Licence annually, in like manner as registers are required to be made under this Act; and it shall be lawful for the Justices of the Peace, assembled at any General or Quarter Sessions of the Peace for the County or Place in which such Licence shall have been granted, as aforesaid, or the major part of them, to withdraw any such Licence, whenever they shall think proper; and the Clerk of the Peace shall therefore give notice to the Registrar, by whom such Licence shall have been registered, as aforesaid, of such Licence being so withdrawn, as aforesaid."

The preceding clauses contain the essence of the Bill, tho' of course many others are added to make them effectual, and are therefore inserted at full length for the information of the Faculty. I trust they have been drawn out with a proper regard to the present Faculty, and with a view to the gradual introduction of a mild and radical amendment. I entertain little doubt that several things mentioned will become objects of keen discussion, as they have a tendency to affect the interests, and prejudices of different persons. Had we now to originate a Medical Establishment for the Empire, I am free to admit, that greater changes and improvements might be recommended; but under existing circumstances and difficulties, perhaps nothing better can be attempted with the prospect of success. It is expressly provided for in the act, that all are to be continued in the capacities which they have been admitted to fill, without being subject to retrospective inquiries. For example, persons having occupied and been admitted *by their brethren* to discharge the duties of regular Physicians, though not graduates in medicine, will by this act become legitimate Physicians, and entitled to act accordingly; I must further observe, that according to the opinion of Chief Justice Mansfield, a Doctor's diploma does not in itself entitle the possessor to practise in the country parts of England. He must be an extra-licentiate of the Royal College of Physicians, or medical graduate of an English University. It is true, the College have no means means of punishing the disobedient, because the statute is unsupported by penalties. This, I conceive, notwithstanding the vaunting of the College, is the reason for suffering the law to remain a dead letter. Doctors in physic, residing in London, are in a different condition; they can be convicted in five pounds the month for practising without the College licence. Still it must be admitted, that the provincial physicians are in a very aukward and humiliating situation. They are only Doctors by courtesy. They cannot, therefore, claim rank, or defend themselves in courts of law. In the cause lately tried at Stafford, before Judge Mansfield, a Physician who graduated in Scotland being grossly abused in his professional capacity, sued for redress, but could obtain none, because he had not complied with the act of Henry VIII. By the provisions of the Bill, Physicians now in being will become Legitimate Practitioners; they will in consequence be entitled to the rank of Doctors, and to protection in all cases. In the same manner, persons from having acted as Surgeons, though they have no diploma, will be made legal, and entitled to the reward of a Surgeon. This is of infinite importance to the present race of surgical practitioners, who, as I formerly observed, can now obtain no legal recompence for professional insults, or any thing performed in surgery, unless they can produce a surgeon's diploma. Such is the irregular and degraded state of medical and surgical practice in England and Wales, where it is ascertained that general learning, personal experience, and the highest medical acquisitions will not avail themselves. It is, however, only reasonable, that the present establishment, from having entered upon their professional duties like their predecessors, without intending any thing improper, should be protected through life; but that no others be suffered to practise until they shall have complied with the Acts of Parliament, and intention of the Crown, in founding the medical Corporations.

Before concluding this long epistle, it will be necessary to remove the objections, and tranquilize the minds of some persons, who have hastily supposed that the proposed regulations will be injurious to the public and subordinate departments, by limiting Physicians, Surgeons, and Apothecaries to their respective lines. It is, indeed, declared in the Bill, that no person shall practise *as* a Physician, Surgeon, or Apothecary, unless he possesses an appropriate authority; but it is no where provided, nor was it, I believe, ever intended, that the same medical men should should be prevented from practising, at pleasure, in all the departments of physic, surgery, or pharmacy; e. g. to practise *as* a Physician, the person must have his name and title inserted in a register, placed on the outer door. He will likewise, as formerly, subjoin his initials to prescriptions. Surgeons and Apothecaries must be registered in the same way. By these means, the several orders of the profession will be fully designated, but not prevented from exercising the different branches of the art. They will only be required to follow them under their respective titles. This is not the obtrusive statement of an uninformed person, it rests *upon high legal authority,* and is therefore deserving of every attention.

I am, &c.

A Friend to a mild and gradual Reform.

*February* 10, 1810.

